# Targeting Oncogenic Gα_q/11_ in Uveal Melanoma

**DOI:** 10.3390/cancers13246195

**Published:** 2021-12-09

**Authors:** Dominic Lapadula, Jeffrey L. Benovic

**Affiliations:** Department of Biochemistry and Molecular Biology, Sidney Kimmel Medical College, Thomas Jefferson University, Philadelphia, PA 19107, USA; dominic.lapadula@students.jefferson.edu

**Keywords:** cancer, G proteins, metastasis, signaling, uveal melanoma

## Abstract

**Simple Summary:**

Uveal melanoma is a deadly form of eye cancer with a high rate of metastasis. Once metastasis occurs, patients are often left with a short survival time, since there is no FDA standard of care for the metastatic disease. Uveal melanoma develops from mutations mainly in proteins involved in the G_q/11_ signaling pathway, which drives pathogenesis. This review article aims to summarize pre-clinical and clinical studies that have attempted to understand and treat the disease by inhibiting the G_q/11_ signaling pathway. We discuss the limited success of treatments focused on downstream targets of the G_q/11_ pathway and evaluate the effectiveness and feasibility of treating the disease by directly inhibiting G_q/11_.

**Abstract:**

Uveal melanoma is the most common intraocular cancer in adults and arises from the transformation of melanocytes in the uveal tract. While treatment of the primary tumor is often effective, 36–50% of patients develop metastatic disease primarily to the liver. While various strategies have been used to treat the metastatic disease, there remain no effective treatments that improve survival. Significant insight has been gained into the pathways that are altered in uveal melanoma, with mutually exclusive activating mutations in the *GNAQ* and *GNA11* genes being found in over 90% of patients. These genes encode the alpha subunits of the hetetrotrimeric G proteins, G_q_ and G_11_, and mutations result in activation of several important signaling pathways, including phospholipase C and activation of the transcription factor YAP. In this review, we discuss current efforts to target various signaling pathways in the treatment of uveal melanoma including recent efforts to target G_q_ and G_11_ in mouse models. While selective targeting of G_q_ and G_11_ provides a potential therapeutic strategy to treat uveal melanoma, it is evident that improved inhibitors and methods of delivery are needed.

## 1. Introduction

Uveal melanoma (UM) is the most common intraocular tumor in adults, and, as the second most common form of melanoma, accounts for approximately 5% of all melanomas. The median age at diagnosis is about 62 years, and risk factors include fair skin, light eye color (green or blue), ocular melanocytosis, dysplastic nevus syndrome, germline BRCA1-associated protein 1 (BAP1) mutations, and welding as an occupation [1,2,3,4,5]. UM arises from melanocytes contained in the choroid, ciliary body, and iris (together known as the uvea) of the ocular cavity, and has a high propensity to metastasize [6,7]. As a result, 36–50% of patients with UM develop metastasis, predominantly to the liver, even when the primary tumor has successfully been eliminated [3,6,8,9,10,11]. While the primary tumor can be successfully treated via enucleation, laser therapy, radiotherapy, or surgical resection, many experts believe that the metastatic spread of UM is unpreventable, as it has likely already taken place by the time the primary tumor is detected [8]. For instance, only 4% of patients show detectable metastasis at the time of diagnosis of the primary tumor, but up to half of all UM patients will develop metastatic disease. This suggests UM develops small, undetectable metastases at an early stage of tumorigenesis, as early as 5 years prior to primary tumor detection, that remain dormant or quiescent for years, classifying UM as a systemic disease requiring some form of systemic therapy [12,13,14]. Once macro-metastasis develops, patients have a poor prognosis with a median overall survival time of less than 1 year as there are currently no effective therapies with a significant impact on survival [6,15,16,17,18,19].

To date, there is no FDA standard of care for metastatic UM. Systemic treatments that have been successful in treating cutaneous melanoma (CM), such as chemotherapy and immunotherapy, have largely failed to produce similar positive results in UM patients. UM response rates to chemotherapy drugs such as dacarbazine, temozolomide, cisplatin, treosulfan, and fotemustine range from 0% to 15%, and no agent has successfully prolonged survival [20,21,22,23,24]. Moreover, immune checkpoint inhibitors of cytotoxic T-lymphocyte-associated antigen 4 (CTLA-4) and programmed cell death-1 (PD-1), which significantly improve survival outcomes for patients with advanced CM, have shown no clinical benefit in patients with metastatic UM [25,26,27,28]. Additionally, while BRAF inhibitors show efficacy in treating CM patients, UM remains unresponsive to similar treatments, despite both malignancies originating from melanocytes derived from the neural-crest [7,29]. These differences may be explained by the observation that UM has a distinctly different etiology from CM. The main oncogenic BRAF and NRAS driver mutations in CM are not typically found in UM [30,31,32,33,34], and while 80% of CM show a UV radiation mutational signature, there is no observed evidence of the same in UM [26,35]. Furthermore, UM has an incredibly low mutational burden compared to CM and most other cancer types [36,37]. With an improved understanding of the genetic differences between cutaneous melanoma and uveal melanoma, there is a clear need for more specific or targeted treatment strategies for UM, as advances in CM treatment will be unlikely to confer the same results in UM.

## 2. Driver Mutations Involved in UM

UM predominantly involves mutually exclusive activating mutations in the *GNAQ* or *GNA11* genes that encode the highly conserved Gα_q_ and Gα_11_ subunits of the heterotrimeric G-proteins [31,38,39]. The introduction of the oncogenic Gα_q/11_ mutants into human or mouse melanocytes results in anchorage-independent growth and gives rise to heavily pigmented tumors in mice [39,40,41]. Interestingly, while about 93% of cases contain oncogenic Gα_q_ or Gα_11_, cases without such mutations often harbor activating mutations in other genes linked to the G_q_ pathway. Activating mutations in *PLCB4*, which encodes for the canonical G_q/11_ effector phospholipase C β (PLCβ), or Cysteinyl Leukotriene Receptor 2 (*CYSLTR2*), a G protein-coupled receptor (GPCR) activator of G_q_, are observed in 2.5% and 4% of cases, respectively [26,36,42,43]. Taken together, it has become clear that aberrant signaling via the G_q_ pathway is the main driver of uveal melanoma.

## 3. Gα_q/11_ Proteins and Their Mutations in UM

Gα_q_ and Gα_11_ proteins are members of the G_q_ subfamily of Gα proteins, are 90% homologous at the amino acid level, and are ubiquitously expressed. To maintain homeostasis in mammalian cells, the activation and deactivation of G-proteins is regulated by tight control over GDP/GTP exchange and GTP hydrolysis rates. Ligand-activated GPCRs, such as CysLTR2, act as guanine nucleotide exchange factors (GEFs) by stimulating the exchange of GDP for GTP on the Gα subunit of the heterotrimeric G-protein. Upon binding to GTP, the Gα subunit changes conformation, dissociates from the Gβγ dimer, and interacts with downstream effectors, such as PLCβ and ARF6 [44,45,46,47,48,49]. Gα proteins contain a Ras-like GTPase domain, wherein important amino acid residues provide the subunit with intrinsic GTP hydrolytic activity, which is accelerated by the interaction of GTPase-activating proteins (GAPs), such as regulators of G protein signaling (RGS). GTP hydrolysis to GDP then terminates the Gα signaling, allowing the subunit to reassociate with Gβγ and return to an inactive state [44,50,51].

The Gα_q/11_ mutations observed in uveal melanoma primarily occur at the Q209 and R183 residues [31,38,39]. Both mutational hotspots are in the GTPase domain and are critical for stabilizing the transition state for GTP hydrolysis. When mutated, the protein loses its GTPase activity and is rendered constitutively active in the GTP-bound state, leading to aberrant proliferative signaling [38,39,47,52,53,54]. Q209 mutations (Gα_q/11_^Q209L/P^) are 13 times more frequent than R183 mutations (Gα_q/11_^R183C^) in UM. While Q209 mutations effectively impede GTPase activity, R183 mutants still retain the ability to hydrolyze GTP, although at a reduced catalytic rate, through interactions with RGS proteins, and still respond to receptor stimulation [39,50,55]. Since Gα_q/11_^Q209^ mutants appear incapable of hydrolyzing GTP even in the presence of RGS proteins, aberrant signaling is increased, leading to a more severe phenotype [52].

## 4. Inhibition of Signaling Pathways Downstream of Activated Gα_q/11_

**Figure 1:**
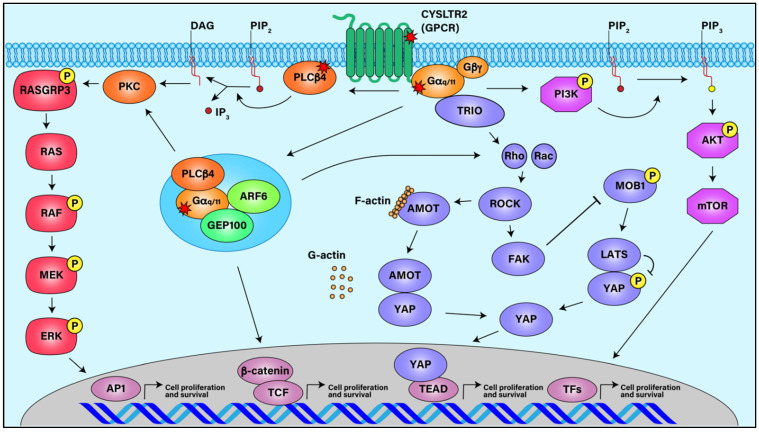
Signaling Pathways in Uveal Melanoma. Activating mutations in GPCR CysLTR2, Gα_q/11_, and PLCβ4 stimulate downstream signaling pathways such as ARF6, MAPK, FAK/YAP, and AKT/MTOR, all of which can lead to UM cell proliferation and survival.

Due to the redundancy of their signaling pathways, oncogenic mutations in either Gα_q_ or Gα_11_ appear to cause similar cellular oncogenic properties leading to the pathogenesis of UM (Figure 1). For example, *GNAQ* and *GNA11* mutations are responsible for the upregulation of mitogen-activated protein kinase (MAPK) pathway, RAF/MEK/ERK, in the absence of BRAF mutations in UM [30,56,57,58]. Knockdown of Gα_q_ in cell lines derived from primary or metastatic UM results in decreased MAPK signaling [38]. Additional G_q/11_ stimulated signaling pathways that promote tumorigenesis have been implicated in UM. Oncogenic Gα_q/11_ leads to aberrant Akt signaling through PI3K/AKT/MTOR and increased activation of small GTPases RhoA and Rac1, which promote cell growth through JNK, p38, and yes-associated protein (YAP)-directed transcription of growth promoting genes [40,41,59,60,61]. To date, most of the efforts to treat UM have mainly been focused on inhibiting the downstream signaling pathways of oncogenic Gα_q/11_ and have provided limited success in improving progression-free survival of UM patients.

### 4.1. ARF6 Inhibition

ADP-ribosylation factor 6 (ARF6) is a small G-protein that has been shown to be an immediate downstream effector of an activated GNAQ/GEP100 complex. ARF6 acts as a proximal node of Gα_q_ signaling to induce all the downstream pathways as well as β-catenin signaling. ARF6 mediates the trafficking of Gα_q_ from the cell membrane to cytoplasmic vesicles and β-catenin to the nucleus. Inhibition of ARF6, by the ARF6 specific small-molecule inhibitor, NAV-2729, reduces uveal melanoma cell proliferation and tumorigenesis in a mouse model [48]. Therefore, ARF6 is considered a potential therapeutic target downstream of oncogenic Gα_q_.

### 4.2. PKC Inhibition

Activated Gα_q_ canonically triggers the protein kinase C (PKC) pathway through diacylglycerol produced by PLCβ activation [44]. Attempts to inhibit PKC in UM have shown promise. The pan-PKC inhibitor, AEB071 (sotrastaurin), at low micromolar concentrations, significantly inhibits the growth of UM cells harboring *GNAQ* mutations, through G1 arrest and apoptosis, and had little effect on UM cells carrying wild-type *GNAQ* [62]. A phase one study of AEB071 resulted in one achieved partial response, while 47% of 118 participants achieved disease stabilization and a median progression-free survival of 15.4 weeks although with some adverse effects [63]. This suggests there is some clinical benefit of PKC inhibition and a new phase one study using the PKC inhibitor LXS196 is currently underway (NCT02601378).

While PKC inhibitors inhibit MAPK signaling and induce G1 arrest in UM cells, they fail to induce tumor regression in xenograft models. As a result, combination treatments of PKC and MEK inhibitors have been attempted and have shown synergistic effects on tumor regression in a UM in vivo model [64]. However, a phase one-b/two study of a PKC and MEK combination treatment of patients with metastatic UM was halted before the beginning of phase two (NCT01801358).

### 4.3. MAPK Pathway Inhibition

The drugs that have been used most often in an attempt to inhibit downstream G_q/11_ signaling in UM are MEK inhibitors, because, similar to CM, MAPK signaling levels are elevated in most UM [57]. Early studies showed MEK inhibitors, such as selumetinib or TAK-733, successfully inhibit UM cell proliferation and viability in vitro [65]. These studies resulted in several clinical trials with various MEK inhibitors, the first of which was a comparison of selumetinib versus chemotherapy treatment, which showed improved progression-free survival (15.9 vs. 7 weeks) but did not significantly improve overall survival (9.1 vs. 11.8 months), while treatment-related adverse events were observed in 97% of patients treated with selumetinib [66]. There is currently recruitment for a new phase one trial to test the effect of intermittent selumetinib administration. The purpose of this study is to test higher drug doses, to more completely block the MAPK pathway and prevent the development of drug resistance mechanisms within the tumor. Intermittent administration of the drug may also reduce side effects (NCT02768766). A phase one study using TAK-733 also showed limited antitumor activity in patients with advanced solid tumors [67]. A recent review analyzed 590 case records from six eligible clinical trials, including the study mentioned above [66]; a phase three study of selumetinib in combination with dacarbazine [68]; phase two studies of selumetinib monotherapy versus temozolomide [69], as well as trametinib, with or without GSK2141795 [70]; a phase one study of trametinib [71]; and a terminated phase one study of AEB071 and MEK162 (NCT01801358). The conclusion is that UM is poorly responsive to MEK inhibitors, regardless of the inhibiting agent and combination partner [72]. The failure of MEK inhibitors to provide meaningful effects on overall survival and tumor growth may be explained by resistance mechanisms that are enabled by the growth factors present in the liver tumor microenvironment [73,74]. However, these results have not deterred investigators from continuing to search for effective combinations with selumetinib. A preclinical study of selumetinib in combination with the ERK inhibitor AZ6197, or the mTORC1/2 inhibitor AZD2014, displayed significant efficacy in two PDX mouse models of UM [75]. Another recent study suggests chloroquine or hydroxychloroquine sensitizes *GNAQ/11*-mutant UM to MEK inhibition by trametinib, through the inhibition of autophagy or lysosome function. This combination treatment synergistically inhibited tumor growth in metastatic mouse models [76].

While MAPK signaling is significantly upregulated in UM, and is often the target of potential therapeutic interventions, the mechanism of how oncogenic Gα_q/11_ activates the MAPK pathway is incompletely understood. The Ras guanyl releasing protein (RASGRP3) is highly expressed in Gα_q/11_-driven tumors, acts as a critical node for ERK activation, and is activated via the PKC δ- and ε-isomers downstream of activated Gα_q/11_ in UM [77,78]. RASGRP3 is required for growth and Ras-MAPK activation in UM cells, as knockdown of the gene transcript leads to decreased MAPK signaling and reduced growth in vitro and in vivo [77,78]. Therefore, RASGRP3 is considered a potential therapeutic target downstream of Gα_q/11_ and represents an additional avenue to MAPK inhibition specific to UM.

### 4.4. PI3K/AKT/MTOR Inhibition

Another activated pathway downstream of oncogenic Gα_q/11_ that has been therapeutically targeted is the PI3K/AKT/MTOR pathway. This pathway is often targeted in combination with MEK or PKC inhibition. One study showed that neither MEK nor PI3K inhibition by trametinib or GSK2126458 alone was sufficient to induce apoptosis in the majority of UM cell lines, but the combination of MEK and PI3K inhibitor treatment resulted in a significant induction of apoptosis in a *GNAQ/11* mutant-dependent manner [79]. Another study co-targeted PI3K and PKC inhibition with BYL719 and AEB071, which showed synergistic inhibition of cell proliferation and apoptotic cell death in UM cells, as well as a significant reduction in tumor growth, in a xenograft model [80]. As a result, a phase one clinical trial of the efficacy and safety of BYL719 and AEB071 drug combination in metastatic UM has begun, but its status is unknown (NCT02273219). Others have targeted AKT in combination with MEK inhibition, which showed induced activation of AMP-activated protein kinase, resulted in the synergistic induction of autophagic cell death in UM cells, and inhibited UM tumor growth in xenograft mouse models [81]. This evidence led to a phase two clinical trial, mentioned previously [70], of trametinib with or without the AKT inhibitor GSK795, which failed to improve progression-free survival compared to selumetinib treatment alone. Additionally, MTOR has also been targeted in UM studies. A phase two clinical trial tested the combination of the MTOR inhibitor everolimus, and the somatostatin receptor agonist pasireotide, in metastatic UM patients. The combination showed little benefit and significant side-effects [82]. A preclinical study found some success in the combination treatment of the MTOR inhibitor everolimus and the PI3K inhibitor GDC0941, which provided increased apoptosis in UM tumors in two PDX models [83]. Another study showed synergistic effects on tumor regression in UM PDX models with a combination of the mTORC1 inhibitor RAD001 and the PKC inhibitor AEB071 [84].

### 4.5. YAP Inhibition

The upregulation of yes-associated protein (YAP) driven by oncogenic Gα_q/11_ in UM is a recent observation [40,41,85]. YAP is a co-transcriptional regulator involved in the cell-growth-regulating Hippo pathway, which, when dephosphorylated, translocates from the cytoplasm into the nucleus, where it associates with TEA domain transcription factor (TEAD) to promote the transcription of growth promoting genes [86,87,88,89]. Studies have shown that knockdown of Gα_q/11_ inactivates YAP, while YAP is required for mutant Gα_q/11_ driven tumorigenesis [40,41]. Further studies indicated that YAP dephosphorylation in UM occurs through the Gα_q/11_ activation of Trio, a guanine nucleotide exchange factor for the small G-proteins, RhoA and Rac1, independent of the Hippo pathway [41]. Additionally, the YAP inhibitor, verteporfin, can inhibit oncogenic Gα_q/11_ UM cell growth in mice [40,41,90]. Although these original studies suggest YAP as a potential therapeutic target of UM, there has since been conflicting studies that suggest YAP may not be as crucial to UM tumorigenesis as originally thought. One study investigating the association between YAP activity and clinical outcome concluded that the effect of YAP on the development, growth, and invasion of UM in patients is less than previously found in experimental studies [91]. Another study investigated the susceptibility of melanoma cell lines to YAP inhibition by verteporfin and found that while most UM cell lines responded in vitro to verteporfin, high risk metastatic UM lines (BAP1-negative) did not. Therefore, the mutational background is an important predictor of response to YAP inhibition by verteporfin, suggesting that not all UM cell lines are susceptible to YAP inhibition [92].

### 4.6. FAK Inhibition

Related to the Hippo pathway and YAP signaling, a recent study showed that Gα_q_ activates focal adhesion kinase (FAK), and that FAK activity is essential for YAP activation and UM cell growth. Using an integrated bioinformatics pipeline, FAK was identified as a candidate synthetic lethal gene with *GNAQ* activation. FAK inhibition, by small molecules VS-4718 or PF562771, suppresses YAP activation in vivo and prevents UM cell growth [93]. Unfortunately, MAPK signaling via oncogenic Gα_q_ provides resistance to FAK inhibition in UM cells. Therefore, a study wherein UM cells were treated with a combination of the MEK inhibitor trametinib and the FAK inhibitor VS-4718 showed a synergistic effect on metastatic UM tumor growth in a mouse model [94]. This study led to a phase two clinical trial, which is currently recruiting metastatic UM patients, testing the efficacy of the combination treatment of the RAF/MEK inhibitor VS-6766 and the FAK inhibitor defactinib (VS-6063) (NCT04720417). Another recent study suggests that PLCβ/PKC activity, but not FAK/YAP, is elevated in UM cell lines as a consequence of Gα_q_ pathway mutations, and that FAK may not be activated independent of PLCβ activation, as previously suggested [95]. This study further illustrates that PKC/MAPK signaling is essential for UM cell proliferation, and that only combined inhibition of PKC and MEK, not FAK and MEK, or FAK and PKC, synergistically reduces cell viability in UM cells [95]. However, this was only shown in a few UM cell lines, and may not be true in every instance of UM, as there is evidence to the contrary, as previously discussed. However, this study, along with the observation that genetic analyses of UM have failed to identify mutations in the FAK or YAP pathways, suggest that FAK or YAP may not be optimal targets for therapeutic intervention in UM [26,96].

## 5. Direct Targeting of Gα_q/11_

**Figure 2:**
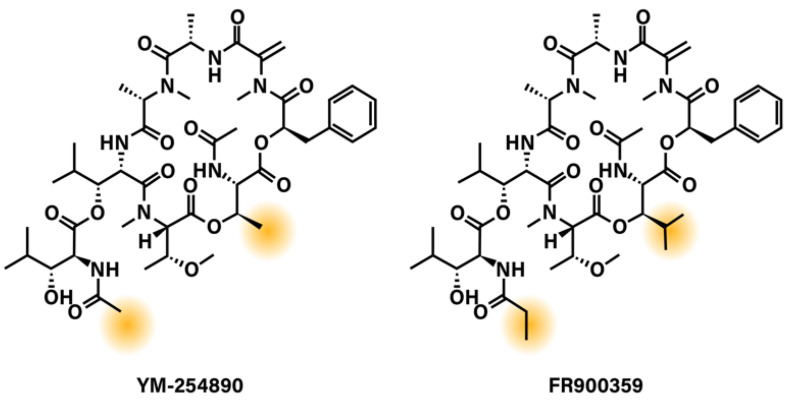
Chemical structures of G_q/11_ depsipeptide inhibitors YM-254890 and FR900359. Highlighted areas show the regions of YM-254890 and FR900359 that differ.

Taken altogether, a potential explanation for these unsuccessful therapies is that oncogenic Gα_q/11_ activates multiple, individually dispensable downstream signaling networks. Inhibition of one, or sometimes two, pathways does not achieve the desired treatment outcome. This raises the possibility that direct inhibition of oncogenic Gα_q/11_ may be an advantageous and promising therapeutic strategy for UM treatment. While there are no current FDA-approved drugs that directly target Gα_q/11_, a few compounds, namely YM-254890 (YM) and FR900359 (FR) (Figure 2), that effectively inhibit Gα_q/11_, have been identified and have shown some promising results in pre-clinical UM studies [97,98].

### 5.1. Short History of G_q_ Inhibitors

YM is a cyclic depsipeptide first isolated from *Chromobacterium* sp. QS3666 in 2003 during a screen for platelet aggregation inhibitors of human plasma [99]. After its isolation, YM was found to reduce the mobilization of intracellular Ca^2+^ by selective inhibition of the G_q_, G_11_, and G_14_ proteins, without affecting the signaling of other G-proteins [55]. G_q_ inhibition by YM was then used as a pharmacological tool to probe G_q_ signaling in cells and mice. Originally it was determined that YM inhibits ADP-induced platelet aggregation (a G_q_-mediated biological response) in vitro, followed by studies that showed that it prevents the formation of thrombosis and neointima in rats and monkeys, and demonstrates antithrombotic and thrombolytic activity [99,100,101,102].

Unfortunately, shortly after its discovery, YM became unavailable to researchers, and in 2012 a worldwide competition for the total synthesis of 1 mg of YM was announced. Due to its complex macrocyclic structure, the total synthesis of YM is not a simple task, but in 2016 the first total synthesis of YM and FR was achieved, albeit with a very low yield [103]. Shortly thereafter, YM became commercially available.

FR, also known as UBO-QIC, is structurally very similar to YM and only differs by the addition of three methyl groups. It was first isolated from the plant *Aridisia crenata sims* in 1988 during an investigation into possible platelet aggregation inhibitors [104]. It has since been shown that FR is synthesized by *Candidatus* Burkholderia crenata, a bacterial symbiont of *Ardisia crenata* Sims [105]. FR is a potent and selective inhibitor of Gα_q/11/14_, has a vasorelaxant effect in rats, and effectively inhibits agonist-promoted airway contraction in human precision-cut lung slices and rats [106,107,108,109]. FR has also been suggested to inhibit Gβγ signaling following the activation of G_i_-coupled receptors [110], although this effect may be due to the involvement of Gα_q_ in Gβγ-mediated activation of PLCβ [111].

FR was commercially available for a short period of time from the König group at the University of Bonn, who isolated it from the dried leaves of *A. crenata* Sims, but the compound is no longer sold. The König group has managed to biosynthesize FR in cultivable *E. coli* by expression of the FR nonribosomal peptide synthetase (*frs*) genes, which are found in *Candidatus* Burkholderia crenata and are responsible for the production of FR in the endosymbiont [112]. The Wollbrett group sought to improve upon the low yield of FR by this method via genetic engineering of *Chromobacterium vaccinii* DSM 25,150, with some success [113]. However, chemical synthesis and biosynthesis methods result in very low yields, leaving purification of the compound from natural sources as the main method of production, as is the case for YM.

### 5.2. Mechanism of Action and Physiochemical Properties of YM and FR

In 2010, the structural basis for inhibition was revealed via an X-ray co-crystal structure of YM bound to a chimeric Gα_i/q_βγ protein complex, showing that YM acts as a guanosine nucleotide dissociation inhibitor (GDI) [114]. The structure revealed that YM binds to a hydrophobic pocket between linker one and switch I of the Gα_q_ subunit, with minimal contact with Gβγ. The region in which linker one and switch I reside is between the helical and GTPase domains and undergoes large conformational changes between the inactive GDP-bound and active GTP-bound conformations. Upon binding of YM, the GDP-bound state of Gα_q_ is stabilized by suppression of the hinge motion of linker one and switch I, resulting in the inhibition of the GDP/GTP exchange, leaving the Gα_q_ protein “locked” in the GDP-bound, inactive conformation [114].

In an effort to characterize FR, a comprehensive 2015 study used molecular dynamics simulations, and a combination of purified proteins and cell-based assays, to show that FR functions similarly to YM as a GDI of Gα_q/11/14_ proteins [107]. This same study determined that FR inhibits Gα_q_ activation in a pseudo-irreversible manner, and that its effects are exceptionally resistant to washout in vasorelaxation and cell-based experiments. Mutagenesis studies established that FR and YM bind to the same hydrophobic pocket of Gα_q_, and that genetically engineered FR-binding sites can be used to create additional Gα proteins, susceptible to FR inhibition, as an investigatory tool [115].

While YM and FR are very similar in their structure and mechanism of action, they do show some biologically distinct activities. FR inhibits Gα_q/11_ with a 3-fold higher potency than YM, and disruption of FR binding to Gα_q_ is more difficult to achieve than the binding of YM [103,115]. This difference in potency may be explained by physiochemical, kinetic, and molecular characteristics that the two very similar compounds do not share. As explained previously, FR differs from YM by the addition of three methyl groups. These differences make FR slightly larger and more lipophilic than YM. A conformational study showed that while FR exists as a single conformer in aqueous solution, YM exists as two different conformers in a major–minor (3:1) ratio. The major form is the biologically active conformer, while the minor represents a conformer that is unable to inhibit Gα_q_ [116]. This suggests that conformational stability is important to the inhibitory potency of these compounds. Additionally, while both compounds display similar association rates (t_1/2_(FR) = 3.6 min versus t_1/2_(YM) = 8.8 min), FR binds pseudo-irreversibly and displays a residence time of 92.1 min, compared to 3.8 min for YM [117]. Molecular docking studies seeking to explain this difference in dissociation rates suggest the additional lipophilic moieties of FR anchor the compound in the binding pocket, similar to “a dowel forming a latch”, while YM lacks those anchor points and may be more readily released from the G_q_ protein [117]. This suggests that even small changes, which seem insignificant within such a large and complex molecule, can lead to differences in potency and potential pharmacological effects.

### 5.3. YM and FR Activity in Uveal Melanoma

Since the discovery of YM and FR, as potent G_q/11_ inhibitors, it was believed that the two compounds could inhibit only wild-type and R183 mutant proteins, but not Q209 mutants, which are almost always the driver mutations in UM. For example, the inhibitory effect of YM on Gα_q_ Q209L and R183C mutants was first tested in HEK293 cells, co-transfected with a serum response element (SRE)-luciferase reporter gene, and either Gα_q_ Q209L or R183C mutant cDNA. This study showed the constitutive activity of the R183C mutant to be completely suppressed by YM, while that of the Q209L mutant was only modestly affected [55]. A comprehensive characterization of FR as a Gα_q/11/14_ selective inhibitor found that it was capable of inhibiting the Q209 mutant Gα_q/11_ [107]. In this study, FR inhibited the growth of a mouse melanoma cell line, Hcmel12, which carries a Gα_q_-Q209L mutation, and is wild type for B-Raf and N-Ras. FR was also found to effectively inhibit nucleotide binding to purified Gα_q_-Q209L protein in vitro, suggesting FR directly binds to the mutant Gα_q/11_ [118]. FR also inhibited the growth and proliferative signaling of UM cells containing oncogenic Gα_q/11_ proteins [118]. Additional studies investigating similar effects of FR emerged around the same time [93,119,120,121] while research involving YM treatment of UM have followed [95,122].

YM and FR show significant inhibitory activity against every mutant Gα_q/11_ variant found in UM in transfected HEK293 cell-based experiments [121,122]. Both compounds selectively inhibit cell growth and promote growth arrest of UM cells harboring oncogenic Gα_q/11_, as well as oncogenic cysteinyl leukotriene receptor 2 (CysLT_2_R^L129Q^) [93,95,118,119,120,121,122]. FR, and likely YM, target both primary and metastatic cells from UM tumors [121]. YM and FR selectively inhibit the essential oncogenic signaling of Gα_q/11_ mutant UM cells. Treatment of UM cells with these G_q/11_ inhibitors abolishes MAPK signaling, suppresses Akt activation, prevents YAP localization to the nucleus, and represses FAK activation [93,95,118,119,120,121,122]. Class one UM cells (wild-type for BAP1) begin to express melan-A at the plasma membrane and gain pigmentation when treated with low nanomolar concentrations of FR [118,119,121]. This suggests FR can induce a redifferentiation of these cancer cells, possibly by decreasing G_q/11_ activity to a level closer to that of a normal melanocyte. It is believed that this FR-mediated redifferentiation is partly the result of restored function to the polycomb repressive complex 2, which plays a role in the differentiation of embryonic stem cells [119,121]. Concentrations of YM and FR at 100 nM and above selectively induce apoptosis and cell death of UM cells, as evidenced by increased caspase-3 and PARP cleavage, as well as an increased population of cells in the sub-G_1_ phase [95,118,119]. These G_q/11_ inhibitors have shown great promise as potential therapeutics in cell-based assays, but are these results recapitulated in vivo?

To address this, there are some recent studies wherein UM mouse models were treated with YM and FR. The first of which showed that FR (0.5 mg/kg) treatment of severe combined immunodeficient (SCID) mice with subcutaneously grafted Gα_q_^Q209P^ UM cells resulted in 72% tumor growth inhibition after 14 days, whereas the growth of xenografts established from B-Raf mutant melanoma cells was unaffected [120]. A second recent study using subcutaneous UM tumor xenografts of Gα_11_^Q209L^ and Gα_q_^Q209L^ UM cells, established in NOD-scid-gamma (NSG) mice, showed that FR inhibited the growth of UM tumors at 0.1 and 0.3 mg/kg, but had no effect on the growth of B-Raf mutant melanoma tumors [121]. However, UM tumors resumed growth 16 days after FR treatment was stopped, suggesting that FR only causes reversible, rather than durable, arrest or regression of UM tumors. ERK phosphorylation in xenograft tumors was only reduced by 30% compared to at least 85% observed in vitro, suggesting FR treatment does not completely suppress aberrant MAPK signaling in vivo. However, FR treatment does provide a therapeutic window in which tumors cells are targeted, while healthy cells maintain the correct physiological G_q/11_ signaling. It is possible the observed incomplete inhibition of MAPK signaling is the result of a resistance or compensatory mechanism at work in these cells, as reverse-phase protein analysis showed an increase of HER3 and activated STAT3 upon FR treatment of UM cells in vitro [118]. Additionally, a recent study showed that YM, similar to FR, inhibited tumor formation in mice with xenografts of Gα_q_^Q209P^ UM cells [122]. Notably, it was observed during this study that YM does not durably suppress MAPK signaling of UM cells in vitro, and that MAPK signature genes begin to rebound after 24 h. This was similarly observed in tumors treated with YM ex vivo. However, a combination strategy of YM plus an MEK inhibitor was able to prevent reactivation and durably suppress MAPK in UM xenograft mice, while the combination also worked synergistically to inhibit tumor growth and promote tumor regression. Taken together, the current in vivo studies suggest G_q/11_ inhibition as a viable therapeutic strategy to treat UM; however, to achieve durable arrest and regression of UM tumors, a combinatorial approach may be warranted.

### 5.4. Feasibility of G_q/11_ Inhibitors as Potential Therapeutics

A recent study that sought to determine if YM and FR are fit for translation, and provides a solid basis for considering future in vivo investigations utilizing YM and FR [123]. It was determined that both YM and FR have high chemical stability under physiological conditions, and both are high clearance compounds in human liver microsomes. However, YM is more stable than FR in human liver microsomes, with short half-lives of 27.3 min and 8.1 min, respectively. Due to the high clearance rate of these compounds, they may not be suitable for systemic application. Importantly, in vivo studies showed that both compounds are only marginally able to cross the blood-brain barrier. However, local administration of these compounds will be preferred, since systemic application can be expected to block G_q/11_ signaling throughout the body. In this regard, the Blumer group determined a LD_50_ of approximately 0.6 mg/kg FR in NSG mice and showed that FR delivered at tolerable doses did not significantly affect heart rate, liver function, or hematopoiesis in xenograft UM models [121].

## 6. Discussion

Uveal melanomas are divided into class one (low metastatic risk) and class two (high metastatic risk) based on gene expression profiling [124]. While almost all UM patients have aberrations in the G_q/11_ pathway, particularly in the Gα_q/11_ proteins, these mutations occur early in tumorigenesis and are not correlated with molecular class or metastasis [31,125]. Class two tumors are strongly associated with an additional BAP1 mutation or deletion that occurs after Gα_q/11_ mutation [5]. This additional mutation increases the metastatic potential of UM, but aberrant G_q/11_ signaling remains as the main driver of tumorigenesis. Recently, the Blumer group has shown that both class one and class two tumor growth is inhibited by G_q/11_ inhibition by FR in UM xenografts [121]. This further strengthens the idea that targeting oncogenic Gα_q/11_ can be a viable therapeutic strategy in treating all subtypes of UM. Targeting oncogenic Gα_q/11_ has the additional benefit, over current monotherapy strategies, of inhibiting the multiple signaling networks downstream of G_q/11_.

While G_q/11_ inhibitors show promise as future therapeutic options, their potential use presents a risk of adverse effects, as they act on both mutated and wild-type Gα_q/11_ proteins, which are ubiquitously expressed and have important physiological functions [126,127,128,129]. Clearly, compounds that can selectively target mutationally activated Gα_q/11_ over wild-type Gα_q/11_ would be ideal as therapeutic agents, but these do not currently exist. However, a growing body of work investigating the important moieties of YM and FR at the molecular level and how these compounds interact with Gα_q/11_, provides valuable insight into the possible synthesis of such a compound. In an effort to synthesize YM, a few simplified YM analogs were synthesized based on the motifs of YM that are thought to be important in maintaining the compound’s stability and making contact with the Gα_q_ protein [130,131]. Its successful synthesis, in 2016, led to the production of many additional analogs used to probe the structure–activity relationship of YM, by modification of key structural elements of the compound [103,116,132,133]. However, all the analogs are less potent than YM, and it has become clear that even small structural changes result in reduced affinity for Gα_q_. Many simplified analogs of FR have also been reported, and while a few selectively inhibit G_q/11_ at a comparable potency to FR, most have failed to inhibit G_q/11_, and none have surpassed the biological activity of FR [116,134,135].

At present, the chemical and synthetic approaches to synthesizing YM and FR have low yields and very complex procedures. Purification of the compounds is the only way to obtain substantial quantities of YM and FR but is time consuming. However, having a method of chemical synthesis provides an avenue to rationally modify these compounds, determine crucial moieties for G_q/11_ inhibition, and possibly design compounds that may be able to more specifically target the oncogenic mutant Gα_q/11_. Some have taken to using biosynthesis methods of creating new YM/FR analogs [136], while others are utilizing feature-based molecular networking techniques to identify new FR analogs produced by other bacterial strains [135,137]. A recent study using tritium-labelled YM and FR compounds in a high throughput competition binding assay discovered novel G_q_ inhibitors, which inhibited G_q_ signaling in recombinant cells and primary murine brown adipocytes, resulting in enhanced differentiation, albeit with significantly less potency than FR [117]. Moreover, the group that first synthesized YM and FR has recently reported a new G_q/11_ inhibitor, GQ127, to be a potent, stable, and safe small molecule. It exhibits good G_q/11_ protein inhibition and antitumor potency in vitro, and significantly inhibits in vivo tumor growth of UM cells [138]. While GQ127 requires significantly higher concentrations compared to YM and FR to observe similar effects on UM cell signaling and tumor growth, it is easily synthesized, displays high oral bioavailability, and shows no obvious side effects in mice. Therefore, it may be an additional structure to build upon for future UM drugs that target G_q/11_.

## 7. Conclusions

Uveal melanoma is a rare melanoma that is biologically distinct from cutaneous melanoma and cannot be treated in a similar manner in the clinic. Despite a high level of success in treating primary UM locally, 36–50% of patients will eventually develop metastasis. To this point, the metastatic disease remains uncurable, and outcomes for patients with UM metastasis are poor [17]. Single agents that target signaling pathways downstream of mutated Gα_q/11_ are not effective, and there is a desperate need for better treatment options. Combinational therapies that co-target multiple pathways are being investigated. The emergence of the G_q/11_ inhibitors YM-254890 and FR900359, and their recent success in treating UM in preclinical studies, suggests that direct inhibition of oncogenic Gα_q/11_ may be a viable approach, or at least provide a therapeutic window to treat the disease more effectively with an additional drug, such as an MEK inhibitor or an inhibitor of survival signals from the liver microenvironment. However, these G_q/11_ inhibitors act on both mutated and wild-type Gα_q/11_ proteins, which raises safety concerns, as G_q/11_ is ubiquitously expressed and has a number of important physiological roles [126,127,128,129]. A local delivery system would be most advantageous, especially one that is able to target the liver, as it is likely that micrometastasis has already formed by the time of initial diagnosis. As we improve our understanding of how these compounds function and interact with G_q/11_, the development of a compound that specifically targets the activated mutant Gα_q/11_ may one day be possible.
